# Report of a Curious Surgical Case

**Published:** 1848-10

**Authors:** John G. Kyle

**Affiliations:** Cedarville, Ohio


### RECORD OF MEDICAL SCIENCE.

*Report of a curious Surgical Case.* By JOHN G. KYLE, M. D., Cedarville, Ohio.—In the spring of 1846, I was called to see ——— Moore, a boy aged two years—had been a very strong, healthy and fleshy child—now weak, much emaciated, and suffering great pain in the bowels—face pale, extremities cold, no appetite, secretions nearly natural, abdomen very tender on pressure, with a swelling or ridge semilunar in shape, commencing on the left and terminating on the right side of the abdomen, and running so nearly to divide the umbilical from the right and left iliac regions—about 14 lines in width, and one or two in height, skin slightly reddened, with the appearance of pointing at the extremity of the swelling on the right side, as if some foreign body was trying to make its way out, being, as yet, however, rather uncertain what direction it should take, in order to reach its intended destination.

The history of the case was, that, fourteen days previous to that time, the boy was badly choked by something, which, after some considerable difficulty he swallowed. After which his parents noticed nothing peculiar for two or three days, when he became fretful and peevish, lost his appetite, and had pain in his bowels. The parents thinking their child had colic, gave anodynes, cathartics and almost every thing else, but finding him growing worse and sinking rapidly, brought him to the village, where I then resided, Roundhead, O., for advice. Being called on, I found the boy in the condition already described. The history of the case with the then present symptoms, led me at once to conclude that the boy had swallowed some solid indigestible substance, and it having become entangled in some fold of intestine, had passed through its coats and was now pointing to the surface, and that an operation would be necessary to relieve the boy. The parents were, however, rather doubtful about the success of an operation, and asked until the next morning to deliberate on the matter, to which I readily assented.

When the morning came, Mr. Moore called and requested me to call and operate on his son, as he believed he would die soon, unless immediately relieved.

The symptoms more aggravated than yesterday—I, in the presence of Dr. A. De Long, L. M. White, Esq., and several other gentlemen, proceeded to operate in the following manner:—The boy being secured, I made an incision ten lines long about equidistant from the umbilicus and ant. sup. spinous process of the right ilium, cutting carefully through the integuments and abdominal muscles; a foreign body could now be felt under the peritoneum, which I punctured with a sharp pointed bistoury, and brought to view a brown corn straw, which I seized with a small pair of forceps, and drew out, applied simple dressings, the wound healed by the first intention, and the boy regained his health in a short time. The corn straw was forked near the middle, measured 33 lines in length, one in diameter, and at the fork near 3 lines across. It had evidently been swallowed by the boy 15 days previous to the operation.

The novelty of the operation, the causes which led to it, and the happy result of the same, are the only apologies the writer has for thus making this case known to his professional brethren.—*Western Lancet.*

